# A Rare Case of Primary Bilateral Adrenal Lymphoma

**DOI:** 10.1155/2017/1251950

**Published:** 2017-07-05

**Authors:** Veeraraghavan Meyyur Aravamudan, Phang Kee Fong, Yang Shiyao Sam, Pavel Singh, Siok-Bian Ng, Gollamudi Satya Pavan Kumar

**Affiliations:** ^1^Department of Advanced Internal Medicine, National University Hospital, 5 Lower Kent Ridge Road, Singapore 119074; ^2^Department of Radiology, National University Hospital, 5 Lower Kent Ridge Road, Singapore 119074; ^3^Department of Pathology, National University of Singapore, National University Hospital, 5 Lower Kent Ridge Road, Singapore 119074

## Abstract

Lymphoma may involve the adrenal glands, but primary lymphoma is rare. Only a few cases have been reported in medical literature. Primary adrenal lymphoma is extremely rare, accounting for <1% of non-Hodgkin lymphomas. We here present a case of a middle-aged female who presented with persistent fever for three weeks. She also reported significant weight loss of more than 10 kgs over the duration of three months. Computed tomography of the thorax and abdomen and pelvis demonstrated bilateral adrenal masses. She underwent short Synacthen test which showed evidence of adrenal insufficiency. She underwent CT-guided adrenal gland biopsy. Histology of adrenal gland biopsy showed features consistent with diffuse large B-cell lymphoma. She was started on R-CHOP chemotherapy and had a good clinical response and remained in complete remission for five months after chemotherapy.

## 1. Introduction

While the majority of lymphomas arise from lymph nodes, up to a quarter develop from extranodal sites [1]. Amongst these, primary adrenal lymphomas are considerably rare, contributing to 3% of all non-Hodgkin lymphomas and interestingly in 70% of the cases both adrenal glands are involved [2]. In this report, we described one such case.

## 2. Case Report

Our patient is a 52-year-old Malay woman who presented with a three-week history of intermittent pyrexia. This was associated with an unintentional weight loss of 10 kg over three months. She has a past medical history of asthma, hypertension, and hyperlipidaemia.

There were no specific localizing symptoms including cough, sputum, haemoptysis, dysuria, abdominal pain, or diarrhoea. There was also no history of travel.

Parameters revealed pyrexia of 40 degrees Celsius and sinus tachycardia of 113 beats per minutes but normal blood pressure. Physical examination was unremarkable with normal heart and breath sounds. Abdomen was soft, nontender with no palpable masses. There was no cervical, axillary, or inguinal lymphadenopathy. There were no neurological deficits and stigmata of liver disease.

Initial investigations showed microcytic hypochromic anaemia with a haemoglobin of 9.8 g/dL and mean cell volume of 75.2 fL. Renal function was unremarkable, but there was a raised lactate dehydrogenase (LDH) level of 2140 U/L. Other blood results are summarised in Table 1.

**Figure 1:**
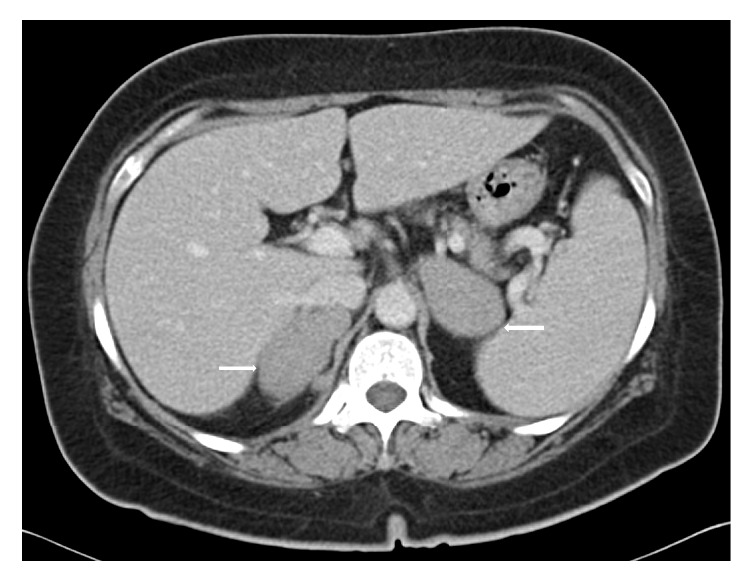
At the time of diagnosis. Imaging studies of patient, CT thorax, and abdomen, and pelvis show bilateral adrenal masses which are homogenous measuring 3.7 × 5.4 × 6.3 cm on the left (78.3 average HU), while the right adrenal gland measures 5.5 × 2.7 × 5.9 cm (72.7 average HU).

**Figure 2:**
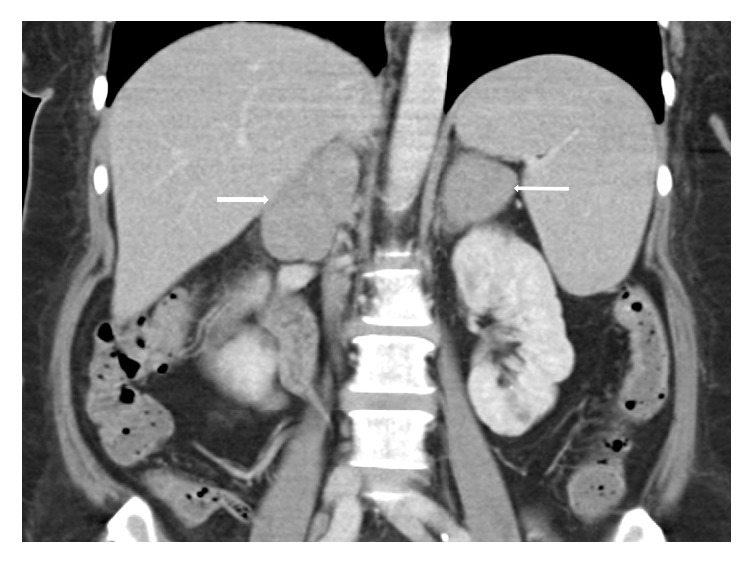
At the time of diagnosis. Imaging studies of patient, CT thorax, and abdomen and pelvis show bilateral adrenal masses which are homogenous measuring 3.7 × 5.4 × 6.3 cm on the left (78.3 average HU), while the right adrenal gland measures 5.5 × 2.7 × 5.9 cm (72.7 average HU).

A computed tomography scan of the thorax abdomen and pelvis was performed, and it showed bilateral homogenous adrenal masses measuring 3.7 × 5.4 × 6.4 cm on the left and 5.5 × 2.7 × 5.9 cm on the right (Figures 1 and 2). There was also a prominent left supraclavicular lymph node measuring 0.8 cm and a few subcentimetre mediastinal lymph nodes measuring up to 0.8 cm.

Early morning cortisol was subsequently found to be low at 242 nmol/L and short Synacthen test with 250 mcg of tetracosactide did not show adequate cortisol response (Table 1). Acid Fast Bacilli stain and culture alongside with Polymerase Chain Reaction for Tuberculosis (TB PCR) of the patient was negative.

A left adrenal biopsy was performed, and histology revealed sheets of large lymphoid cells with a prominent intravascular growth pattern. The neoplastic cells were positive for CD20, CD79A, MUM1, BCL6, and BCL2. There was no expression for CD10, consistent with a nongerminal centre B-cell origin. MYC was positive in 70% of tumour cells and Ki-67 proliferation index was 90%. CD5 was positive; Cyclin D1 and SOX11 were negative, which also excludes mantle cell lymphoma. Bone marrow biopsy did not show any marrow involvement (Figure 6).

The patient was reviewed by a Hematologist, and the decision was made to manage her as for Stage 4 Diffuse Large B-Cell Lymphoma (DLBCL) in view of organ involvement. Her Eastern Cooperative Oncology Group (ECOG) status was zero, and International Prognostic Index (IPI) was one because of raised lactate dehydrogenase. She was treated with six cycles of R-CHOP and four cycles of intrathecal methotrexate chemotherapy.

**Figure 3:**
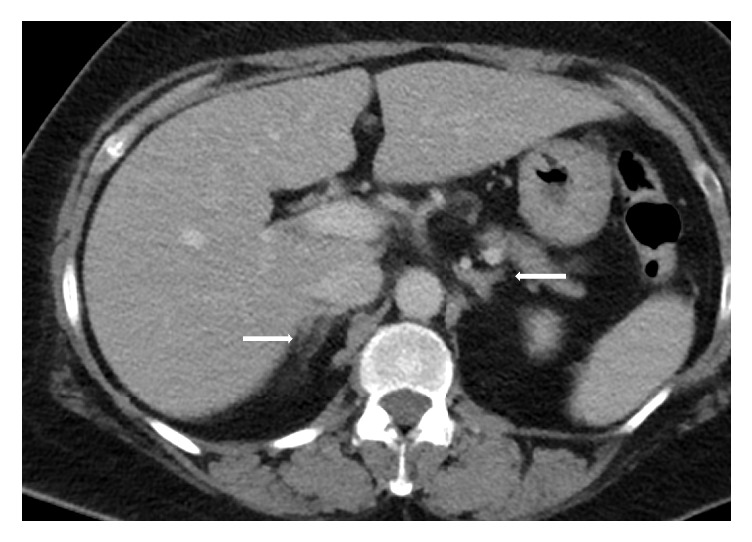
At the end of treatment. Imaging studies of the patient after having chemotherapy show interval regression of bilateral adrenal glands with both adrenal glands returning to normal size.

Remarkably, repeat computer tomography scan after four cycles of R-CHOP chemotherapy showed interval regression of bilateral adrenal masses with nearly normal adrenal glands (Figure 3). The left enlarged supraclavicular and mediastinal nodes remained stable in size.

**Figure 4:**
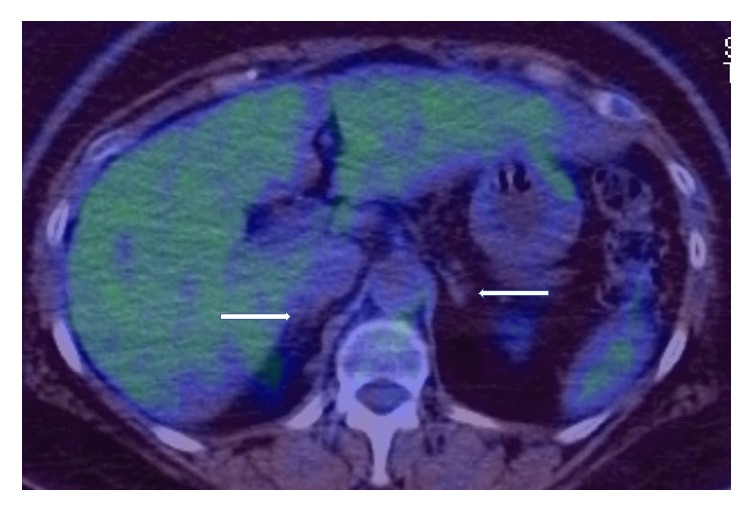
At the end of treatment. Posttreatment PET-CT scan showing normal sized adrenal glands (white arrows) with no significant FDG activity.

**Figure 5:**
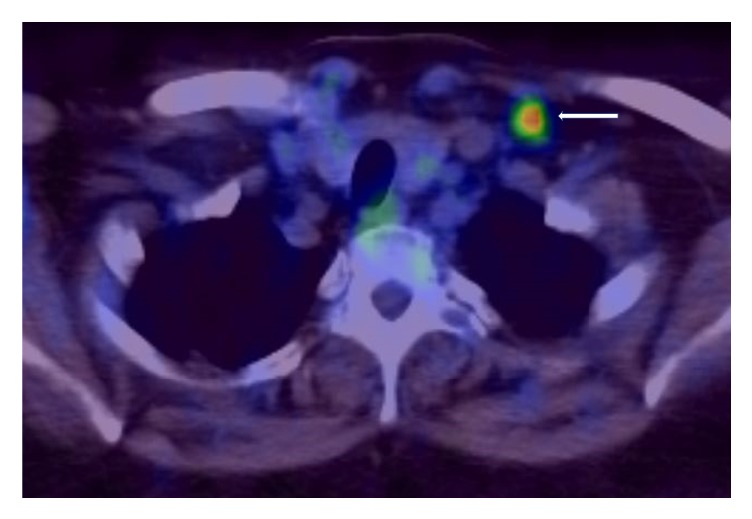
At the end of treatment. Posttreatment PET-CT scan showing a borderline enlarged left supraclavicular lymph node (white arrow) with moderate to intense FDG activity (SUVmax 8.4). In the absence of any significant adenopathy elsewhere in the body, this was deemed to be of reactive nature.

**Figure 6:**
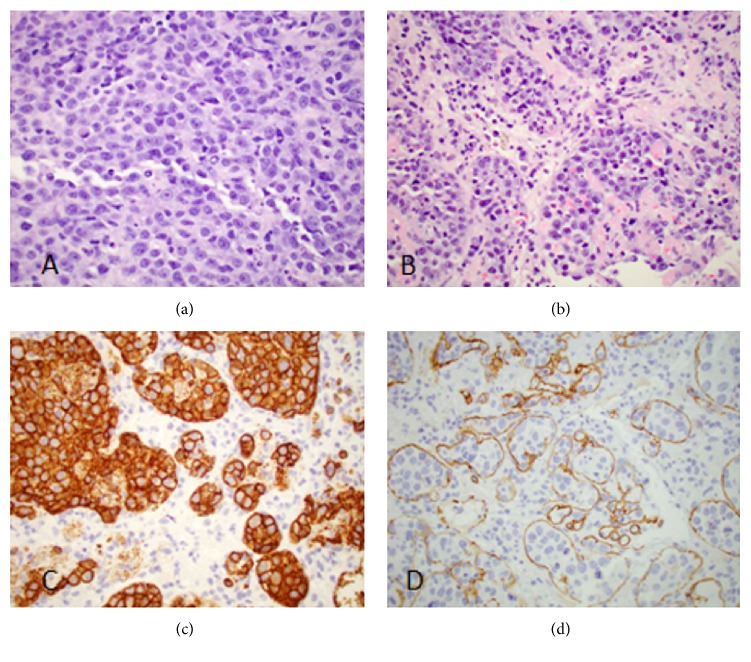
Histology images. The neoplastic lymphoid cells showed large vesicular nuclei ((a) H&E, original magnification ×600) and prominent intravascular growth pattern ((b) original magnification ×400). They were positive for CD20 ((c) original magnification ×600). CD34 stain ((d) original magnification ×400) highlighted vascular channels containing neoplastic lymphoid cells, confirming the intravascular growth pattern.

A Positron Emission Tomography (PET) scan six weeks after completion of both R-CHOP and intrathecal methotrexate revealed no fluorodeoxyglucose (FDG) update in the chest, abdomen, or pelvis and the enlarged left supraclavicular lymph node showed mild-FDG avidity that was smaller in size compared to previous computed tomography scans. This was discussed in a multidisciplinary tumour board, and it was decided to consider this as a reactive lymph node (please see Figures 4 and 5).

She remained in complete remission five months after chemotherapy and have since returned to work. However, repeat Synacthen test showed persistent adrenal insufficiency, and she remained on hydrocortisone replacement therapy. She is being monitored closely by a Hematologist and an Endocrinologist.

## 3. Discussion

Primary adrenal lymphoma is a typically highly aggressive malignancy. As exemplified by our patient, most cases are usually diffuse large B-cell lymphomas and bilateral involvement is frequently observed [3]. Age, tumour size, adrenal insufficiency, lactate dehydrogenase level, and performance status of the patient can significantly influence prognosis [4, 5].

Diagnosis can be established with the help of biopsy and histological examination [6]. The European Society of Endocrinology [7] recommends imaging studies and hormonal assessment for bilateral adrenal masses first. This is because there are many differential diagnoses ranging from metastases from a different primary lymphomas or bilateral pheochromocytoma. In addition, there remains a possibility of cooccurrence of different entities, resulting in the necessity of separately characterising each lesion. Biopsy is recommended for hormonally inactive lesions; lesions which cannot be concluded as benign by imaging and histological diagnosis alter the management. Surgical intervention, however, is suggested to be individualised according to various factors including the patient's age, comorbidities, and preferences.

In our case, we elected a percutaneous needle biopsy according to patient preference. It was, therefore, reassuring that posttreatment scans showed resolution of both adrenal masses. In accordance with the works of Ichikawa et al., our patient responded well to a rituximab-containing regimen combined with intrathecal methotrexate, suggesting that this combination is effective against adrenal lymphomas [8].

## 4. Conclusion

Bilateral adrenal lymphoma remains a rare form of non-Hodgkin lymphoma and care must be taken in ruling out other causes of bilateral adrenal masses. Subsequent surgical interventions need to be individualised. Although our experience suggests that a rituximab-containing regimen with intrathecal methotrexate is effective, larger studies should ideally be carried out to validate this treatment modality.

## 5. Learning Points

Commonly bilateral adrenal masses are usually due to metastases from malignant tumours arising from lungs, breast, or colon.

Primary DLBCL affecting both adrenals is a rare phenomenon [9].

Diffuse large B-cell lymphoma (DLBCL) is the most common histological subtype.

## Figures and Tables

**Table 1:** Initial blood test results.

Test	Results	Unit	Reference interval
White blood cells	6.45	×10^9^/L	3.40–9.60
Red blood cells	3.87	×10^12^/L	3.70–9.60
Haemoglobin	9.8	g/dL	10.9–15.1
Mean Cell Volume	75.2	fL	80.0–95.0
Mean corpuscular haemoglobin	25.3	pg	27.0–33.0
Mean corpuscular haemoglobin concentration	33.7	g/dL	32.0–36.0
Haematocrit	29.1	%	32.7–44.4
Platelets	150	×10^9^/L	132–372
Mean platelet volume	9.3	fL	8.7–12.2
Red cell distribution width	18.2	%	11.4–14.8
Sodium	137	mmol/L	135–145
Potassium	4.4	mmol/L	3.5–5.0
Chloride	101	mmol/L	95–110
Carbon dioxide	22	mmol/L	22–31
Creatinine	66	umol/L	50–90
Urea	4.9	mmol/L	2.0–6.5
Glucose	7.2	mmol/L	4.0–7.8
Albumin	34	g/L	38–48
Bilirubin, total	7	umol/L	5–30
Bilirubin, conjugated	1	umol/L	0–5
Aspartate aminotransferase	64	U/L	10–50
Alanine aminotransferase	48	U/L	10–70
Alkaline Phosphatase	162	U/L	40–130
Lactate dehydrogenase	2140	U/L	250–580
Calcium, total	2.22	mmol/L	2.15–2.55
C-reactive protein	65	mg/L	0–10
Iron	3.3	umol/L	8.8–27.0
Ferritin	1249	ug/L	10–120
Transferrin	201	mg/dL	200–360
Total iron binding capacity	52	umol/L	52–94
Iron saturation	6	%	15–50
Thyroxine, free	12.9	pmol/L	8.0–20.0
Thyroid stimulating hormone	1.59	mIU/L	0.45–4.5
Early morning cortisol	242	nmol/L	123–623
Adrenocorticotropic hormone (ACTH)	3.1	pmol/L	0.0–10.2
*Synacthen test*			
Cortisol at 0 min	150	nmol/L	
Cortisol at 30 min	191	nmol/L	
Cortisol at 60 min	209	nmol/L	
